# Adjuvant radiotherapy after radical cystectomy for patients with muscle invasive bladder cancer: a phase II trial

**DOI:** 10.1186/s12885-017-3302-9

**Published:** 2017-05-02

**Authors:** Valérie Fonteyne, Piet Dirix, Sara Junius, Elke Rammant, Piet Ost, Gert De Meerleer, Martijn Swimberghe, Karel Decaestecker

**Affiliations:** 1 Department of Radiation-Oncology, Ghent University Hospital, De Pintelaan 185, Ghent, Belgium; 2 Department of Radiation-Oncology, Iridium Cancer Network, Ghent, Belgium; 3 Department of Radiation-Oncology CH-M/AMPR, Mouscron, Belgium; 4 Department of Radiotherapy and Experimental Cancer Research, Ghent University, Ghent, Belgium; 5 Department of Urology, Ghent University Hospital, Ghent, Belgium

**Keywords:** Muscle invasive bladder cancer-adjuvant radiotherapy-toxicity

## Abstract

**Background:**

Neo-adjuvant chemotherapy followed by radical cystectomy with extended pelvic lymph node dissection is considered to be the treatment of choice for patients with muscle invasive bladder cancer (MIBC). Despite this aggressive treatment the outcome is poor and ultimately, 30% of the patients with ≥pT3 tumors develop a pelvic recurrence. We hypothesize that postoperative adjuvant external beam radiotherapy (EBRT) might prevent local and lymph node recurrence and improve disease free- and overall survival as loco-regional recurrence is linked to the development of distant metastasis.

**Methods:**

We plan to perform a multicentric prospective phase two study including 76 patients. Eligible patients are patients with MIBC, treated with radical cystectomy and presenting with ≥1 of the following characteristics:

- Pathological (p)T3 stage + presence of lymphovascular invasion on pathological examination
- pT4 stage
- <10 lymph nodes removed
- positive lymph nodes
- positive surgical margins

Patients will have a ^18^F–FDG PET-CT to rule out the presence of distant metastasis prior to EBRT. A median dose of 50 Gy in 25 fractions is prescribed to the pelvic lymph node regions with inclusion of the operative bladder bed in case of a positive surgical margin. Patients with suspected lymph nodes on PET- CT can still be included in the trial, but a simultaneous integrated boost to 74Gy to the positive lymph nodes will be delivered. Blood and urine samples will be collected on day-1 and last day of EBRT for evaluation of biomarkers. The primary endpoint is evaluation of acute ≥Grade 3 intestinal or grade 4 urinary toxicity, in case of a neo-bladder reconstruction, within 12 weeks after EBRT. Secondary endpoints are: assessment of QOL, late RTOG toxicity, local control, disease free survival and overall survival. Biomarkers in urine and blood will be correlated with secondary survival endpoints.

**Discussion:**

This is a prospective phase 2 trial re-assessing the feasibility of adjuvant radiotherapy in high-risk MIBC.

**Trial registration:**

The Ethics committee of the Ghent University Hospital (EC2014/0630) approved this study on 31/07/2014.

Trial registration on Clinicaltrials.gov (NCT02397434) on November 19, 2014.

## Background

Muscle invasive bladder cancer (MIBC) is the ninth most frequently diagnosed cancer and ranks 13th in terms of causes of death worldwide [1]. The incidence of bladder cancer increases steadily with age. Due to improved life expectancy, the number of patients diagnosed with MIBC is expected to increase in the future.

A radical cystectomy combined with an extended pelvic lymph node dissection (ePLND) is considered to be the treatment of choice. Cisplatin-based neo-adjuvant chemotherapy improves survival outcomes and is recommended for patients with cT2-T4a N0–1 M0 MIBC [2, 3]. Despite this multimodality treatment the outcome did not altered significantly over the last 30 years [4] and remains poor with 5-years overall survival rates ranging between 50 [5] and 60% [6] to 65% [7]. For patients with ≥pT3 MIBC the 5-year overall survival even drops to as low as 22% [8]. Others have reported somewhat better results with a 5-year bladder cancer specific survival of 31 and 48% for T4 and T3 MIBC patients respectively [6].

Besides the high rate of systemic recurrences, loco-regional control is equally important. Approximately 30% of patients with ≥pT3 tumors develop a pelvic recurrence [8–10]. For instance, in a contemporary monocentric analysis from a high-volume, tertiary center focusing on 334 pT3–4 pN0–1 MIBC patients, 31% of patients developed a pelvic recurrence, 34% of which had loco-regional only failure. Chemotherapy, whether immediately postoperative (adjuvant) or delayed at time of recurrence, might improve the outcome of patients with MIBC as was suggested in a recent meta-analysis [11]. Unfortunately, these results are based on very inhomogeneous data. The conclusions of this meta-analysis are not considered to be robust and it remains unclear which patients really benefit from it [12]. Generally, neo-adjuvant chemotherapy is preferred, which does not interfere with this trial. Currently, there are also several trials already running or being initiated looking at the use of adjuvant immunotherapy with MIBC. While this approach looks promising, eventual adjuvant immunotherapy does not exclude adjuvant radiotherapy. In fact, there might be an additional benefit in the combination [13].

The rationale for adjuvant radiotherapy also reflects that attempts to salvage patients with pelvic failures are rarely successful [14]. Indeed, 1- and 2-years survival for patients developing a local recurrence after cystectomy is only 8 and 3% respectively, with a median survival of <4 months [15]. For patients with lymph node recurrence prognosis is somewhat better, but nevertheless still disappointing with reported 1- and 2 years survival of 42 and 11% respectively [15]. Moreover, pelvic recurrences are often debilitating with pain, lymphedema and venous thrombosis as known morbidities. If patients with local or lymph node recurrence are treated with external beam radiotherapy (EBRT), 1-year survival is significantly improved from 2% (no EBRT) to 27% [15]. Apparently, improving loco-regional control can impact survival in MIBC. We hypothesize that an earlier implementation of pelvic EBRT i.e. in the immediate postoperative (i.e. adjuvant) setting, will prevent local and pelvic nodal recurrence and improve disease free- and overall survival as loco-regional recurrence is linked to the development of distant metastasis [16, 17].

Little is known about the timing of EBRT combined with radical cystectomy in MIBC. Preoperative EBRT can prevent intra-operative seeding of tumor cells and sterilizes microscopic extensions in the perivesical tissues. However, a meta-analysis of six randomized trials showed no clear benefit for preoperative irradiation [18]. Adjuvant EBRT also has the advantage of dealing with microscopic cells along with a better identification of patients who are really at increased risk of developing local recurrence based on histological information of the primary tumor. Already two decennia ago, adjuvant EBRT was tested in a prospective randomized trial and resulted in a 20% increase in 5-year disease free survival (DFS) [19, 20]. These results were supported by a non-randomized controlled Radiation Therapy Oncology Group trial [21]. Also in the era of modern surgery it is suggested that adjuvant radiotherapy improves outcome. Shariat et al. found that, although a limited number of patients, adjuvant radiotherapy was independently associated with lower disease recurrence (HR: 3.3, 95% CI: 2.2–4.9; *p* < 0.001) and improved disease specific survival (HR: 2.4, 95% CI: 1.6–3.7; *p* < 0.001) while in the same study the impact of adjuvant chemotherapy on similar outcomes was less clear [6]. Despite those encouraging results, severe intestinal toxicity rates hampered the enthusiasm to use adjuvant EBRT, in fact till now [19, 21]. However, the technology that was used in the above-mentioned trials is outdated and conclusions concerning toxicity do not apply to the current technologies such as intensity-modulated radiotherapy (IMRT) and volumetric arc therapy (VMAT) and image guided radiotherapy [22–24]. Therefore, it is reasonable and even desirable to reconsider the use of adjuvant EBRT in properly selected MIBC patients.

This “proper” selection should be based on clinical, pathological, radiological and biological predictors for local and locoregional failure. Well-known clinical and pathological predictors are T-stage, lymph node status [6, 25], presence of lymphovascular invasion [6] and surgical margin status [10].

To select those MIBC patients who will benefit most from postoperative EBRT it is important to first rule out distant metastasis. So far, conventional imaging techniques fail to detect recurrences or metastasis from bladder cancer at an early stage. Although only few studies have evaluated the potential role of ^18^F–FDG-PET-CT in the loco-regional staging of patients with MIBC, specificity and negative predictive values mounts to >90% rendering it an interesting tool in the staging of MIBC patients, although its use is currently recommended only within clinical trials [26]. ^18^F–FDG-PET-CT has also been studied in the recurrent setting. Although the evidence is limited it has been suggested that a good diagnostic performance is obtained with ^18^F–FDG-PET-CT in the recurrent setting of MIBC [27]. By implementing ^18^F–FDG-PET-CT prior to the start of adjuvant radiotherapy we aim to exclude patients with distant metastasis as these patients are less likely to benefit from this treatment approach.

Additionally, circulating biomarkers can be used to identify patients who are at increased risk for local and pelvic nodal relapse for whom this multimodality therapy is presumed to be most beneficial. Biomarkers will be evaluated to determine if they can help in selecting those patients most likely to benefit from adjuvant radiotherapy.

## Methods/design

This study is approved by the Ethics committee of the Ghent University Hospital (EC2014/0630) and is registered on clinicaltrials.gov (NCT02397434). Patients with high risk (see below) of developing local or pelvic nodal recurrence after radical cystectomy with ePLND will be offered adjuvant EBRT within a multicentric prospective phase II trial.

### Objectives

- *Primary endpoint*
  - ○ Acute toxicity defined as toxicity occurring during or within 3 months following adjuvant EBRT and scored by:
    - ▪ Acute Radiation Therapy Oncology Group (RTOG) small and large intestine toxicity [28] and Common toxicity criteria version 4.0 (CTC v4.0) [29]
    - ▪ Acute RTOG [28] and CTC v4.0 [29] bladder toxicity for patients with a neobladder only
- *Secondary endpoints*
  - ○ Quality of life (QOL) scoring using the EORTC QLQ-C30 supplemented with QLQ-BLM30 prior to, at the end and 1 month after adjuvant EBRT, then 3-monthly during the first year and 6-monthly up to 2 years.
  - ○ Assessment of quality-adjusted-life-years with the EuroQol classification system (EQ-5D) [30]. A written consent to use this system was obtained from the EuroQol Group Foundation.
  - ○ Assessment of erectile function using the International Index of Erectile Function (IIEF) questionnaire [31] in males and sexual function in females [32].
  - ○ Assessment of voiding pattern using the International prostate Symptom Score (I-PSS) [33] and International Consultation on Incontinence Modular Questionnaire Urinary Incontinence (ICIQ-UI) Short Form [34] for patients with a neobladder.
  - ○ Late toxicity due to radiotherapy will be scored using RTOG [28] and CTC v4.0 [29] scoring systems. Late toxicity is defined as toxicity lasting more than 3 months after cessation of adjuvant EBRT, or starting more than 3 months after the end of adjuvant radiotherapy.
  - ○ Local control rate defined as absence of recurrence within the pelvis. This will be evaluated on pelvic CT scans performed on predefined time-points (see below) or in case of suspicion of recurrence.
  - ○ DFS defined as survival without evidence of disease recurrence (neither local recurrence, recurrence in lymph nodes or distant metastasis) and is calculated from date of radical cystectomy till diagnosis of local, regional or distant recurrence on CT scan at predefined time-points or in case of suspicion of recurrence.
  - ○ Bladder cancer specific survival calculated from date of radical cystectomy till death due to MIBC.
  - ○ Overall survival calculated from date of radical cystectomy till death of any cause.
  - ○ Serum samples and urine samples after cystectomy will be collected and stored in a biobank. Biomarkers will be evaluated on all samples in duplicate and correlated with DFS and bladder cancer specific survival.

### Inclusion criteria

Eligible patients are patients with M0-MIBC, treated with radical cystectomy and ePLND with ≥1 of the following characteristics:

- pathological (p)T3-MIBC with presence of lymphovascular invasion on pathological examination
- pT4 -MIBC
- <10 lymph nodes removed
- presence of pathological positive lymph nodes
- positive surgical margins

and

- WHO performance state 0–2
- Age ≥ 18 years old
- Willing and able to provide a signed informed consent

### Exclusion criteria

- Presence of distant metastasis at time of inclusion
- Previous pelvic irradiation
- Presence of any psychological, familial, sociological or geographical condition potentially hampering compliance with the study protocol and follow-up schedule
- Presence of a second primary tumour other than accidental finding of completely removed prostate cancer, non-melanoma skin tumour or tumour diagnosed ≥5 years ago

### Evaluation and inclusion

**Fig. 1:**
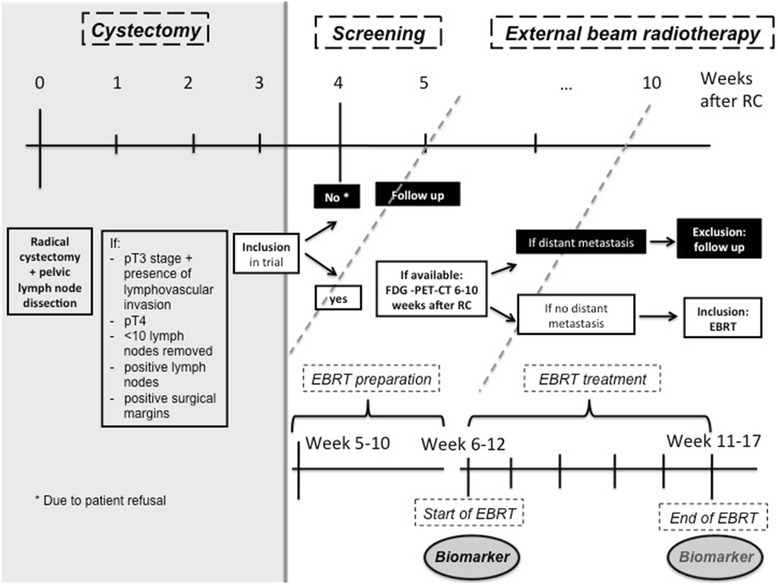
Overview of study flow chart. Abbreviations: p: pathological; EBRT: external beam radiotherapy; RC: radical cystectomy

A flow chart presenting the different steps from inclusion until treatment is presented in Fig. 1. EBRT should start during week 6–12 after radical cystectomy.

### Intervention

A ^18^F–FDG-PET-CT is recommended but not obligatory within 4–10 weeks after radical cystectomy to rule out distant metastases.

All patients receive a planning CT on week 5–10 after radical cystectomy. Planning CT is performed in supine position with 3 mm CT slice thickness from the lung top till mid femur.

The target volume comprises the pelvic lymph node areas at risk in all cases. This stems from the observation that most recurrences are in the pelvic sidewall region so that the central pelvis, and the cystectomy bed in particular, might reasonably be spared treatment unless margins are involved [8, 11]. The lymph node regions at risk are the lymph nodes located along the common, internal and external iliac artery, the lymph nodes in the obturator fossa and the presacral nodes. Suspicious lymph nodes on ^18^F–FDG-PET-CT imaging are delineated separately. All delineated lymph node regions at risk are then summed to a structure named as Lnn. The clinical target volume (CTV)_Lnn is then created using an isotropic expansion of 2 mm around the Lnn. The planning target volume (PTV)_Lnn is created using a 5 mm margin around the CTN_Lnn.

The bladder bed is only included in the radiation field if there is a positive surgical margin [8, 14]. Pre cystectomy imaging as well as information obtained from the surgical report and anatomopathological evaluation is used for delineation of the bladder bed.

A median dose of 50 Gy is prescribed to the pelvic lymph nodes ± bladder bed and is delivered in 25 fractions, 5 times a week. A simultaneous integrated boost up to 70 Gy (corresponding to a normalised iso-effective dose of 74 Gy in 2 Gy fractions calculated with an α/β of 10 [35]) to the positive lymph nodes, detected on (^18^F–FDG-PET-)CT, is prescribed. VMAT is used. The treatment is delivered on a linear accelerator using 6–18 MV photons and multileaf collimation [23, 24]. Patient positioning during treatment is controlled by daily cone beam CT.

### Follow up

Patients are followed weekly during therapy, 1 month after therapy and 3- monthly thereafter. After the end of radiotherapy and at each follow up visit a standard blood control (including sedimentation, erythrocytes, leucocytes (including formula), thrombocytes, creatinine, electrolytes, liver parameters, alkaline phosphatase) is performed. In asymptomatic patients imaging (CT thorax/abdomen/pelvis) will be performed 3–6-monthly after the end of radiotherapy during the first year and 6-monthly thereafter up to a period of 5 years and yearly thereafter or until progression.

### Statistical analysis

#### Sample size

We plan to perform a multicentric prospective phase 2 study in which 76 patients will be enrolled. With this sample size there is 95% likelihood that no more than 25% ± 10% [21] of the patients will develop severe toxicity (i.e. grade ≥ 3 toxicity requiring hospitalization and/or surgical re-intervention) after adjuvant radiotherapy. This number takes into account 5% percentage of missing data.

### Data analysis

The primary endpoint is acute toxicity defined as any toxicity from start of adjuvant EBRT up to 3 months following adjuvant EBRT.

The incidence of late toxicity will be recorded. Actuarial risk estimates for developing late toxicity will be calculated using Kaplan Meier analysis.

QOL will be evaluated pre-radiotherapy, at the end of radiotherapy and 1 month after radiotherapy. Thereafter a 3 monthly evaluation will be performed during the first year and 6-monthly up to 2 years after radiotherapy.

DFS, bladder cancer specific survival and overall survival will also be calculated using Kaplan Meier actuarial analysis. Survival times are defined from the date of radical cystectomy until an event or last follow up.

## Discussion

For patients with MIBC neo-adjuvant chemotherapy and radical surgery is standard of care [3]. Unfortunately the outcome remains poor for high-risk MIBC patients and the overall survival hasn’t improved significantly over the past years. Further efforts to improve the outcome of these patients are therefore mandatory. Previous trials showed that adjuvant radiotherapy can improve DFS at the cost of severe toxicity [9–20]. The latter limited the enthusiasm to propose adjuvant radiotherapy to patients with high-risk MIBC. In the last decade, major technological advancements in radiotherapy delivery have been realized, resulting in a better coverage of the target volume while sparing more normal tissue (mainly small bowel) and in a more precise delivery of radiation. VMAT is such a technique that can significantly reduce late toxicity [23, 24]. Furthermore, better insights in the pattern of local relapse, based on large surgical data [8–10], enable us to tailor and restrict the radiation field to the area that is at highest risk of developing recurrence. Based on these observations there is a renewed interest in reevaluating the place of adjuvant radiotherapy in those MIBC patients who are at increased risk of developing disease recurrence [36]. The aim of this prospective phase 2 trial is to support the hypothesis that with modern radiotherapy toxicity of adjuvant radiotherapy after radical cystectomy is acceptable.

## Abbreviations

c: Clinical stage
CT: Computed tomography
CTC: Common toxicity criteria
CTV: Clinical target volume
DFS: Disease free survival
EBRT: External beam radiotherapy
EORTC: European Organisation for Research and Treatment of Cancer
ePLND: Extended pelvic lymph node dissection
EQ-5D: EuroQol classification system
Gy: Gray
ICIQ_UI: International Consultation on Incontinence Modular Questionnaire Urinary Incontinence
IIEF: International Index of Erectile Function
IMRT: Intensity-modulated radiotherapy
Lnn: Lymph nodes
MIBC: Muscle invasive bladder cancer
p: Pathological stage
PTV: Planning target volume
QOL: Quality of life
RTOG: Radiation Therapy Oncology Group
VMAT: Volumetric arc therapy
